# Presurgical naso-alveolar molding paired with cheiloplasty to treat median cleft lip deformity in holoprosencephaly

**DOI:** 10.1080/23320885.2020.1753518

**Published:** 2020-04-17

**Authors:** Satoshi Takagi, Ayumu Tsukamoto, Yoshihisa Kawakami, Sachio Tamaoki, Hiroyuki Ohjimi

**Affiliations:** a Department of Plastic and Reconstructive Surgery, School of Medicine, Fukuoka University, Fukuoka, Japan; b Section of Orthodontics, Department of Oral Growth and Development, Fukuoka Dental College, Fukuoka, Japan

**Keywords:** Holoprosencephaly, median cleft, presurgical nasoalveolar molding, cheiloplasty

## Abstract

We report median cleft lip in an infant girl with lobar-typed holoprosencephaly who underwent presurgical naso-alveolar molding and subsequent cheiloplasty. At seven months postoperatively, we observed an upper lip with natural cupid-bow-shape formed with a nasal dome and two nostrils separated with reconstructed columella, which were maintained for eight years.

## Introduction

Holoprosencephaly refers to congenital defects occurring when the forebrain does not divide, or fails to divide sufficiently, into the left and right hemispheres during early embryogenesis. Although holoprosencephalic fetuses usually die in the first trimester—leading to miscarriages—few infants with mild lobar holoprosencephaly are born [1]. These infants present with forebrain anomalies and facial malformations including medial cleft lip and shortened interorbital distance between the eyes.

One main characteristic of the median cleft lip is the considerable lack of tissue in the facial midline. In some cases, the primary palate and the columella are completely missing, making reconstruction extremely difficult. Various reconstruction methods have been reported based on ‘straight-line closure of the upper lip’ [2–4]. Herein, we report a case of holoprosencephaly with median cleft lip in an infant girl who underwent cheiloplasty and presurgical naso-alveolar molding (PNAM). To our knowledge, this is the first case of PNAM for holoprosencephaly in the literature.

## Case description

**Figure 1.**
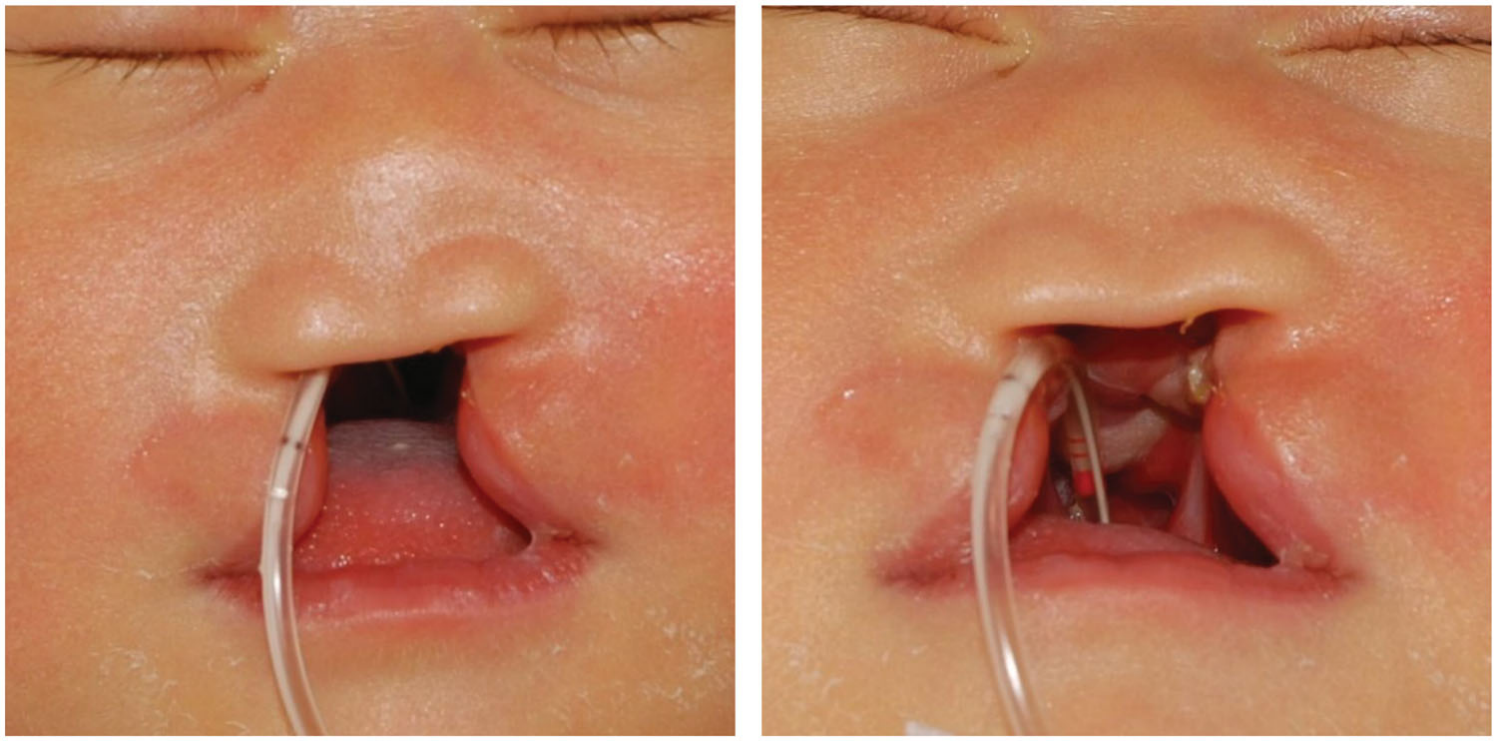
Median cleft lip and flatted nasal dome in a 7-days old girl with lobar-type-holoprosencephaly.

**Figure 2.**
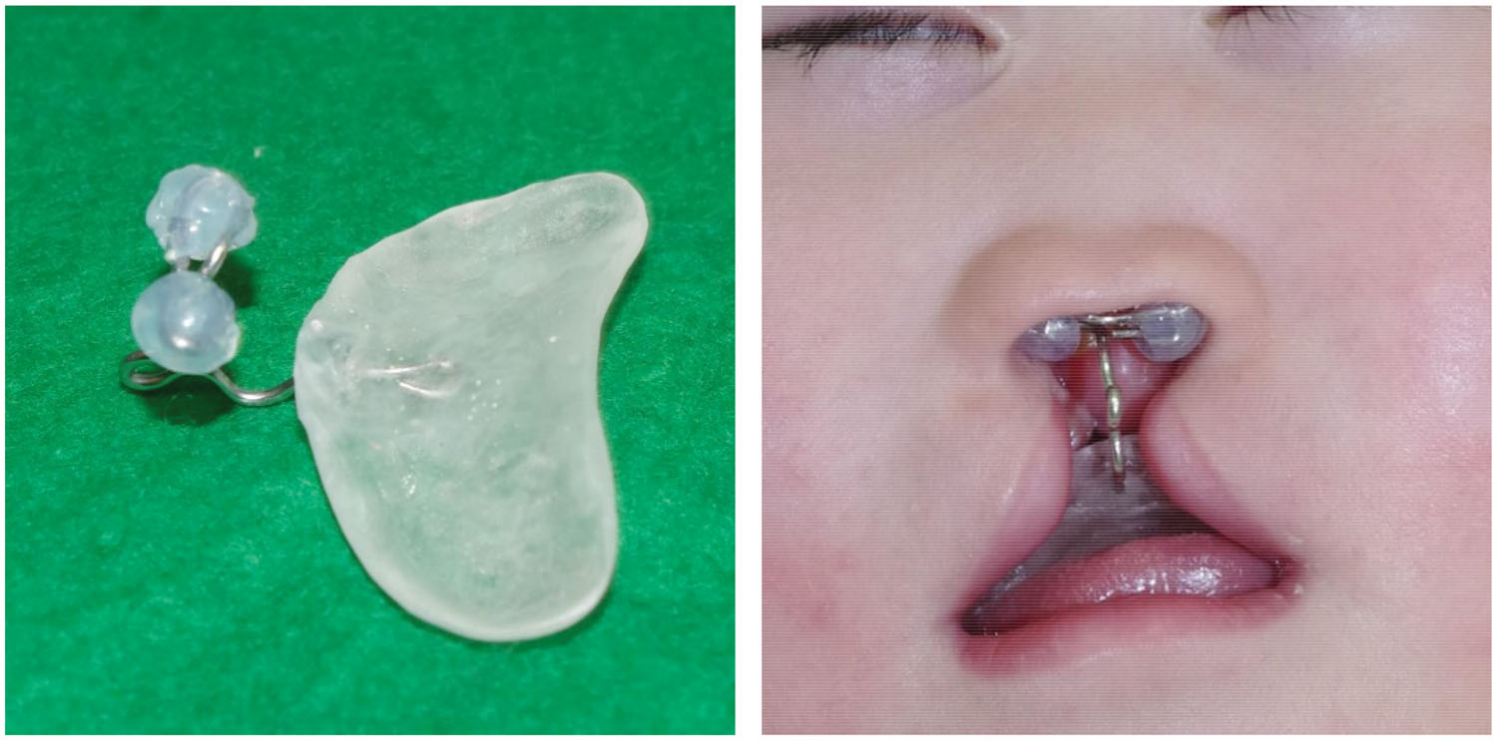
Naso-alveolar molding plate and its silicone globes, which support the nasal dome in the upward and forward direction.

An infant girl with lobar-type-holoprosencephaly was referred to us regarding her median cleft lip in the first postnatal week. Her premaxilla, columellar, and nasal septal cartilage were completely absent, and the nasal dome was flatted without any support (Figure 1). Since we intended to correct and mold the alar nasal cartilage, we performed PNAM for her when she was 14 days old. Frontally, on a customized resin plate, a curved steal wire and two silicone globes were attached to support the nasal dome up and forward (Figure 2). The patient showed signs of growing up, although her growth was slow and her naso-alveolar shape and size changed gradually. We followed up with her monthly and, if needed, we remade the resin plate or changed the curvature of steel wire of PNAM plate.

**Figure 3.**
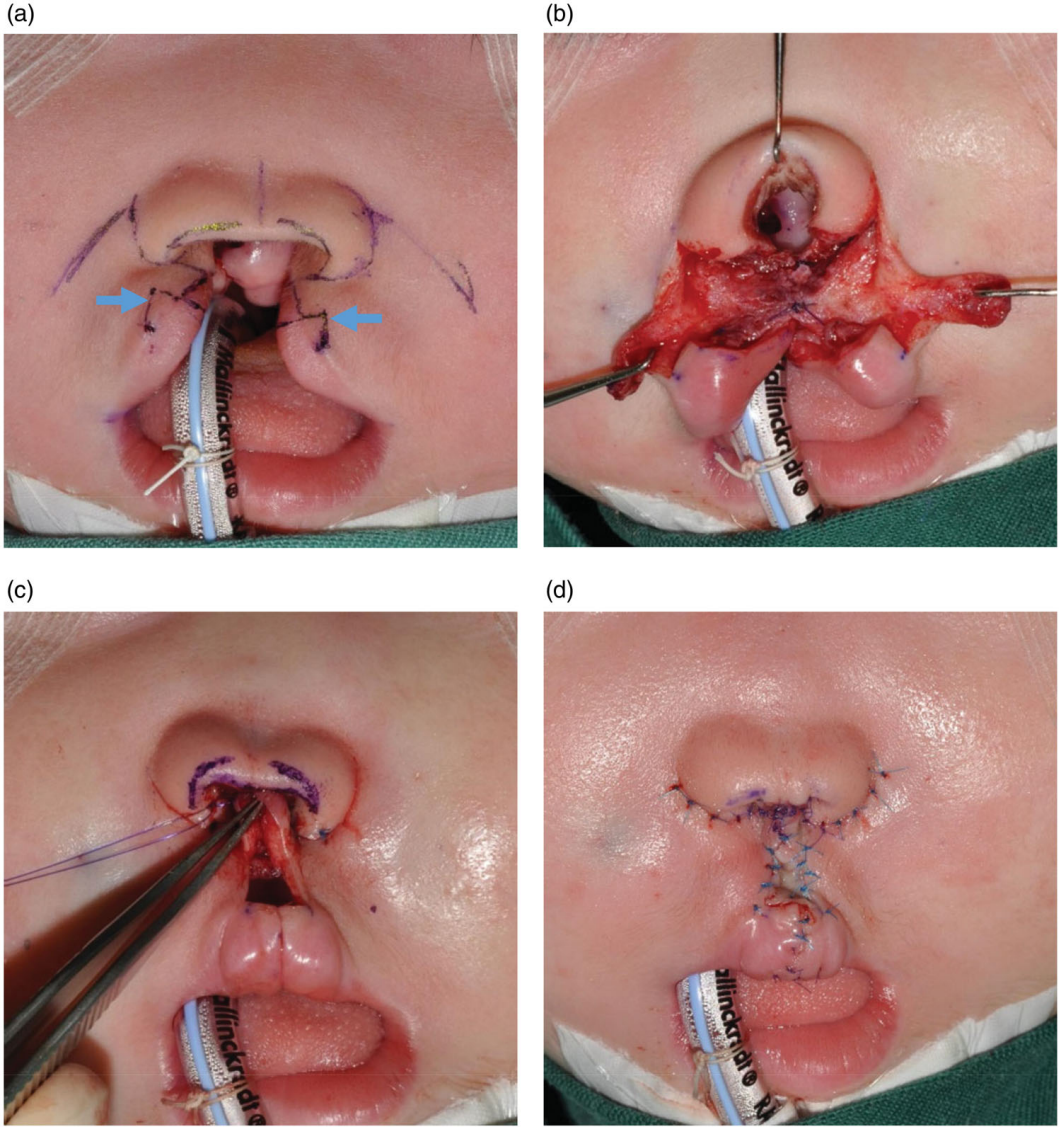
Cheiloplasty performed after 18 months of PNAM. (a and b) Columellar reconstruction was performed with local flaps. Arrow: small white lip portions with lateral vermillion. (c and d) A skin graft was applied on the philtrum.

Finally, at her age of 18 months, after estimating the tolerance to surgery, a definitive cheiloplasty was performed, which involved closing the lateral vermillion elements together at the labial center with small white lip portions (Figure 3), which finally induced the natural growth of cupid bow-shaped lips. Columellar reconstruction was also performed with local flaps from the upper lip. The two pedicled flaps were transposed and fixed columnar to the middle of the nasal dome. A piece of skin graft was applied on the philtrum. No peri- or postoperative complications occurred; the blood loss was minimal and the postoperative course was unremarkable.

**Figure 4.**
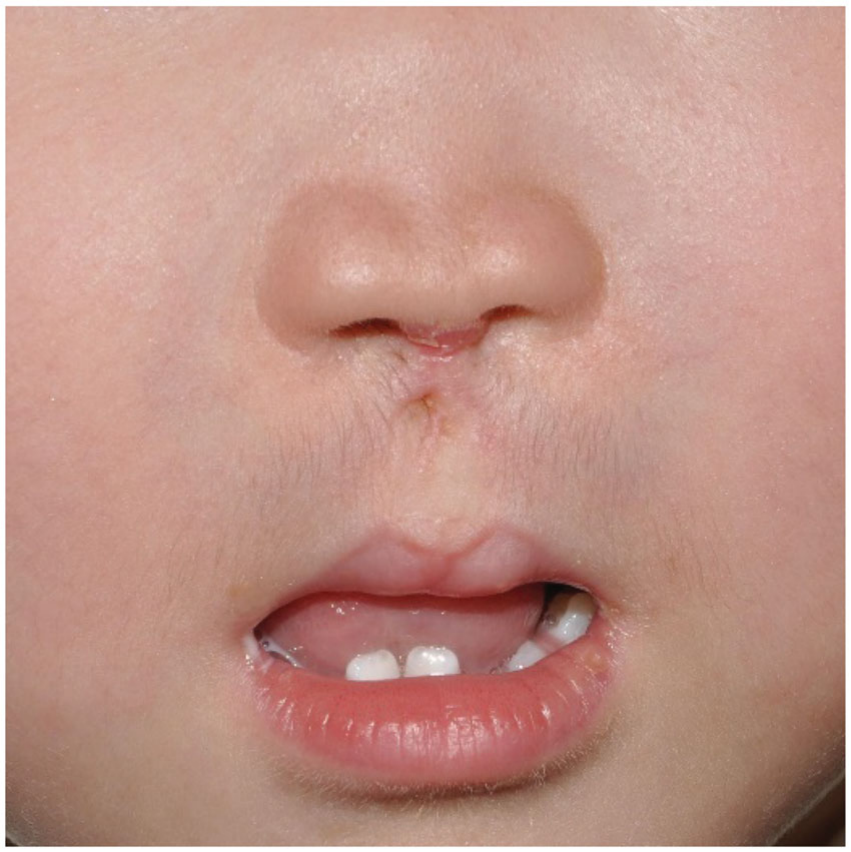
At seven months postoperatively.

**Figure 5.**
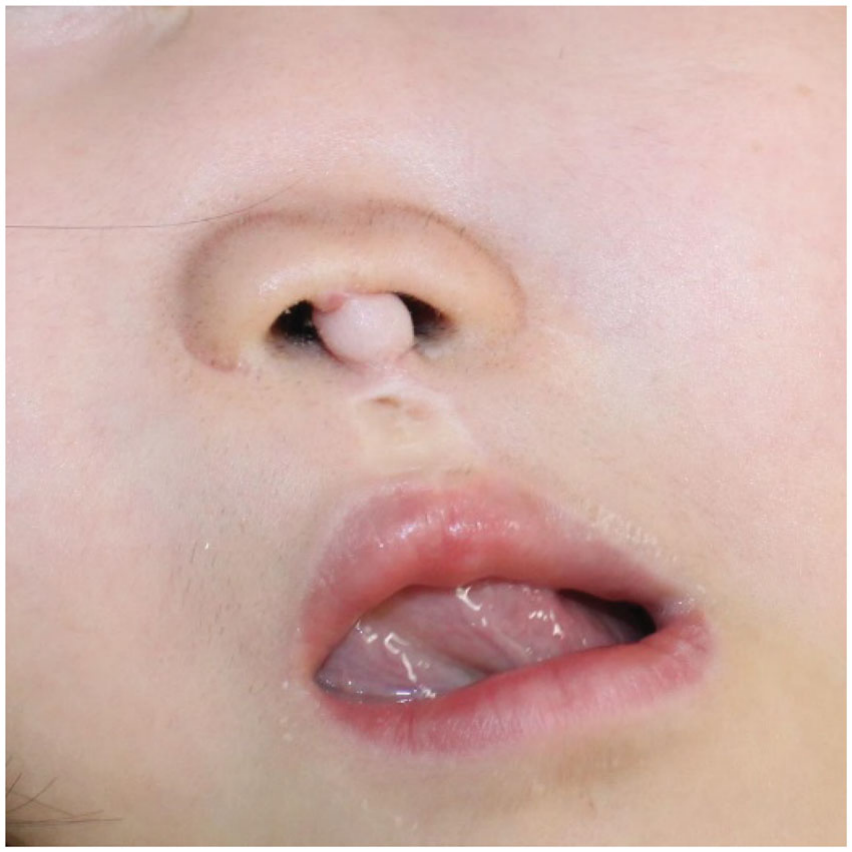
Defined cupid-bow of the lips, cupid peaks, and the columellar strut supporting the nasal dome after the eight-year follow-up period.

Seven months later, a well-formed cupid-bow lip with cupid peaks was observed, which was maintained for eight years postoperatively (Figures 4 and 5). We also noted that the two nostrils had enlarged and the reconstructed columella as well.

## Discussion

Holoprosencephaly is a malformation sequence with a basic feature of impaired midline cleavage of the embryonic forebrain. Among clinical features, facial anomalies are the most easily perceived in the patient, which have varying severities according to the extent of brain damage [5]. Besides facial abnormalities, holoprosencephaly can associate with complications including neurocognitive impairment, seizures, diabetes insipidus, autonomic instability, recurrent respiratory infections, and major organ dysfunction. Moreover, the life expectancy may be shortened based on the severity. Still, some parents may choose reconstructive surgery for the facial deformities in their infants. When the patients can well tolerate the general anesthesia, surgical intervention, and perioperative care under general surgical conditions, medical practitioners should recommend cheiloplasty as an effective treatment option since it enables relatively higher developmental abilities in the patients than without. This not only helps them achieve speech development but also helps in creating better social interactions.

In holoprosencephaly-related median cleft lip, the premaxillary structure is completely missing or critically rudimentary. As limited skin and soft tissue volume was available for cheiloplasty, it was quite challenging to reconstruct the lip and nose into a fine structure that forms the cupid bow, philtrum ridge, philtrum dimple, or labial tubercle. The simplest cheiloplasty should involve a straight approximation of the lateral upper lip [2–4]. But if a straight scar on the upper lip center is present, the shape of the philtrum structure cannot be determined. For the philtrum reconstruction, Sadove *et al.* applied a free skin graft [6]. We used skin grafts in our surgical procedure for restoring the philtrum shape; however, it was limited to the region covering the cephalad area and the philtrum dimple. A small white lip approximated with a lateral upper lip segment can fill the caudal area up to the philtrum dimple and simultaneously push the red lip downward, which can mediate the formation of a proper cupid-bow shape.

The columellar reconstruction was also significant; it was performed using the local flaps from the alar margin or upper lip by following previously reported methods [7,8]. However, those flaps are insufficient to firmly support the radically aberrant alar tissue and appropriately lift the depressed nasal tip. Hence, we believe that the presurgical application of the PNAM plate aided in shape-correction of the alar tissue, including the alar cartilage, to some extent and minimized the depressed nasal tip. In our case, the nasal septal cartilage was completely deficient and the only support for the nasal dome was the reconstructed columellar soft tissue, which might only weakly support the nasal dome. Hence, we decided to follow-up carefully with the patient to further observe the changes in the nose during her growing years and her overall growth.

## Conclusion

We attempted to apply presurgical naso-alveolar molding (PNAM) in addition to cheiloplasty for our infant patient with holoprosencephaly-associated median left clip, which has never been reported in the literature. Successful treatment outcomes were obtained after a 7-year follow-up period.

## References

[1] Barr M, Jr., Cohen MM. Jr. Holoprosencephaly survival and performance. Am J Med Genet. 1999;89(2):116–120. 10559767

[2] Gawrych E, Janiszewska-Olszowska J, Walecka A, et al. Lobar holoprosencephaly with a median cleft: case report. Cleft Palate Craniofac J. 2009;46(5):549–554. 19929095 10.1597/08-059.1

[3] Hendi JM, Nemerofsky R, Stolman C, et al. Plastic surgery considerations for holoprosencephaly patients. J Craniofac Surg. 2004;15(4):675–677. 15213551 10.1097/00001665-200407000-00027

[4] Morita N, Morita Y, Taenaka Y, et al. Two cases of single-stage lip and nostril reconstruction in holoprosencephaly. Int J Oral Maxillofac Surg. 2011;40(8):862–865. 21470822 10.1016/j.ijom.2011.02.025

[5] El-Hawrani A, Sohn M, Noga M, et al. The face does predict the brain–midline facial and forebrain defects uncovered during the investigation of nasal obstruction and rhinorrhea. Case report and a review of holoprosencephaly and its classifications. Int J Pediatr Otorhinolaryngol. 2006;70(5):935–940. 16280170 10.1016/j.ijporl.2005.09.020

[6] Sadove AM, Eppley BL, DeMyer W. Single stage repair of the median cleft lip deformity in holoprosencephaly. J Craniomaxillofac Surg. 1989;17(8):363–366. 2592577 10.1016/s1010-5182(89)80107-6

[7] Livaoğlu M, Imamoğlu Y. Columellar reconstruction: a new technique for median cleft lip repair. J Craniofac Surg. 2017;28(2):506–507. 28005651 10.1097/SCS.0000000000003300

[8] Saad MN, Barron JN. Reconstruction of the columella with alar margin flaps. Br J Plast Surg. 1980;33(4):427–429. 7426824 10.1016/0007-1226(80)90108-3

